# Developing transmission line equations of oxygen transport for predicting oxygen distribution in the arterial system

**DOI:** 10.1038/s41598-018-23743-2

**Published:** 2018-03-29

**Authors:** Fei Yan, Wen-Tao Jiang, Zhi Xu, Qing-Yuan Wang, Yu-Bo Fan, Ming Zhang

**Affiliations:** 1 Laboratory of Biomechanical Engineering, Department of Applied Mechanics, Sichuan University, Chengdu, 610065 China; 2 Department of Biomedical Engineering, The Hong Kong Polytechnic University, Hong Kong, China; 3 Institute for Disaster Management and Reconstruction (IDMR), Sichuan University — The Hong Kong Polytechnic University, Chengdu, 610065 China; 4 School of Biological Science and Medical Engineering, Beihang University, Beijing, 100191 China

## Abstract

The oxygen content in the arterial system plays a significant role in determining the physiological status of a human body. Understanding the oxygen concentration distribution in the arterial system is beneficial for the prevention and intervention of vascular disease. However, the oxygen concentration in the arteries could not be noninvasively monitored in clinical research. Although the oxygen concentration distribution in a vessel could be obtained from a three-dimensional (3D) numerical simulation of blood flow coupled with oxygen transport, a 3D numerical simulation of the systemic arterial tree is complicated and requires considerable computational resources and time. However, the lumped parameter model of oxygen transport derived from transmission line equations of oxygen transport requires fewer computational resources and less time to numerically predict the oxygen concentration distribution in the systemic arterial tree. In this study, transmission line equations of oxygen transport are developed according to the theory of oxygen transport in the vessel, and fluid transmission line equations are used as the theoretical reference for the development. The transmission line equations of oxygen transport could also be regarded as the theoretical basis for developing lumped parameter models of other substances in blood.

## Introduction

Oxygen plays a significant role in the physiological status of the tissues in a human body. The cellular metabolism in tissues necessitates the oxygen derived from the oxygen transfer in microcirculation, where the oxygen transfer from the blood to the tissue consists of diffusion from the arteriole, capillary, and venule to the tissue^1^. Oxygen transport in the arteries is the most important method of supplying oxygen and nutrients to the tissue. In terms of the oxygen transport in a vascular segment, the oxygen concentration distribution is determined by the principle which is based on the mass balance of oxygen transport in the segment, and this principle states that the difference of the oxygen concentration between inflow and outflow from the segment and the oxygen diffusion from the blood to the arterial wall dominate the oxygen concentration distribution in the corresponding segment^2,3^. The oxygen concentration difference between inflow and outflow mainly depends on the hemodynamic response in the vessel. Oxygen diffusion from the blood to the arterial wall is the main component of oxygen consumption in the arterial tissue. An abnormality in the oxygen tension (PO_2_) distribution might contribute to hypoxia in the arterial wall^4^, which could further contribute to the development of atherosclerotic plaque^5–8^.

Most studies on the oxygen distribution in the human body concentrated on the experimental measurement and numerical prediction of the PO_2_ in microcirculation and large arteries. Numerous measurements of the arterial PO_2_ were performed through using invasive procedures^9–12^, and there were few noninvasive methods of measuring the oxygen concentration in the systemic arteries. Thus, several three-dimensional (3D) computational fluid dynamic (CFD) simulations coupled with oxygen transport have been performed to investigate the oxygen distribution in the arterial blood and the arterial wall in more detail^4,12–15^. Most of these studies have primarily focused on the PO_2_ distribution in local regions and did not consider the systemic distribution of oxygen concentration from the perspective of the whole circulatory system.

In fact, the oxygen distribution in the systemic arterial tree is supposed to be acquired from a 3D CFD simulation coupled with oxygen transport. This simulation is complicated and requires considerable computational resources and time in terms of systemic circulation. It is currently almost impossible for most of the research groups and clinical measurements to perform a 3D simulation of the systemic arterial tree.

The lumped parameter model of blood flow based on the fluid transmission line model is a useful tool in predicting the systemic distribution of blood flow and pressure in the arterial tree. Noordergraaf *et al*.^16^ developed a lumped parameter model of the whole circulatory system, including the left ventricle, and compared the results with the human longitudinal ballistocardiogram; The comparison between the results of the lumped parameter model and the measured data indicated that the lumped parameter model was reliable in obtaining the behaviors of a real circulatory system^17^; Lacourse *et al*.^18^ applied the lumped parameter model of the upper circulatory system to simulate stenosis at different locations of the arterial tree and investigate the effects of atherosclerosis on the systemic distribution of hemodynamic parameters. The lumped parameter model of blood flow could be used not only to determine the effects of cardiovascular diseases on the systemic hemodynamics of the circulatory system, but also to predict the hemodynamic responses of clinical interventions. Liang *et al*.^19,20^ developed a lumped parameter model of the entire circulatory system based on patient-specific clinical data to predict the effects of the Fontan operation on the systemic hemodynamic data. The systemic distribution of the oxygen concentration in the arterial tree was not considered in most of the lumped parameter models. A simplified mathematical expression that included the hemoglobin concentration was applied in the lumped parameter model to calculate the blood oxygen concentration from a systemic circulation perspective^21,22^. However, the effects of hemodynamics on the oxygen concentration distribution were neglected in the numerical investigations. Hemodynamics plays a significant role in oxygen transport in the arteries and should be considered for an accurate calculation^2,13,15^. Different inflow velocities cause the changes in the convective flow and affect the oxygen concentration distribution^23^. More specifically, increasing the inflow velocity would enhance the convective flow, and thus tend to decrease the oxygen transport resistance and increase the oxygen concentration^4^.

In the present study, transmission line equations of oxygen transport were developed regarding the transmission line equations of blood flow. Hemodynamics is treated as a significant factor for calculating the oxygen concentration in these transmission line equations. The convection term of oxygen transport is mainly affected by blood flow, and oxygen convection dominates the oxygen concentration distribution in blood. The resulting lumped parameter model of oxygen transport, which is established based on the proposed transmission line equations of oxygen transport, should be valuable for predicting the oxygen concentration distribution in the whole circulatory system.

## Theoretical Considerations

### Integrated form of the oxygen transport equation

The mass transport equation in cylindrical coordinates is applied as follows in the derivation of the oxygen transmission line equations:

1 $$\frac{\partial C}{\partial t}+\frac{{v}_{r}}{r}\frac{\partial }{\partial r}(rC)+\frac{{v}_{\theta }}{r}\frac{\partial C}{\partial \theta }+{v}_{z}\frac{\partial C}{\partial z}=\frac{{\rm{\Gamma }}}{\rho }[\frac{1}{r}\frac{\partial }{\partial r}(r\frac{\partial C}{\partial r})+\frac{1}{{r}^{2}}\frac{{\partial }^{2}C}{\partial {\theta }^{2}}+\frac{{\partial }^{2}C}{\partial {z}^{2}}]+S,$$

where *r*, *θ*, and *z* are the radial, angular, and axial coordinates, respectively; *C* is the solute concentration; Γ and *ρ* are the diffusion coefficient of the solute and the fluid density, respectively; *v*_*r*_, *v*_*θ*_, and *v*_*z*_ are the components of the fluid velocity in the *r*, *θ*, and *z* directions in the cylindrical coordinate system, respectively; and *S* is the source term. The source term *S* is assumed to be zero in terms of oxygen transport in the blood vessel, while *v*_*r*_ and *v*_*θ*_ in a fully developed flow are usually assumed to be zero in the numerical simulation of blood flow. Subsequently, equation (1) can be written as follows:

2 $$\frac{\partial (rC)}{\partial t}+{v}_{z}\frac{\partial (rC)}{\partial z}=\frac{{\rm{\Gamma }}}{\rho }[\frac{\partial }{\partial r}(r\frac{\partial C}{\partial r})+\frac{{\partial }^{2}(rC)}{\partial {z}^{2}}].$$

We then integrate each term of equation (2) over the cross-section of the blood vessel and use the Leibniz rule (equation (3)) for differentiating an integral to rewrite the integrals.

3 $$\frac{\partial }{\partial t}{\int }_{\alpha (t)}^{\beta (t)}f(x,t)dx={\int }_{\alpha (t)}^{\beta (t)}\frac{\partial f(x,t)}{\partial t}dx+\{f[\beta (t),t]\frac{\partial \beta (t)}{\partial t}-f[\alpha (t),t]\frac{\partial \alpha (t)}{\partial t}\}$$

Thus, equation (2) is transformed into:

4 $$\frac{\partial ({\int }_{0}^{R}2\pi rCdr)}{\partial t}+{v}_{z}\frac{\partial ({\int }_{0}^{R}2\pi rCdr)}{\partial z}=\frac{{\rm{\Gamma }}}{\rho }[{\int }_{0}^{R}2\pi \frac{\partial }{\partial r}(r\frac{\partial C}{\partial r})dr+\frac{{\partial }^{2}({\int }_{0}^{R}2\pi rCdr)}{\partial {z}^{2}}],$$

where *R* is the radius of the vascular lumen. The oxygen content of the whole cross-section (M) is given by the expression $M={\int }_{0}^{R}2\pi rCdr$. Moreover, the first term on the right-hand side of equation (4) could be rewritten as

$${\int }_{0}^{R}2\pi \frac{\partial }{\partial r}(r\frac{\partial C}{\partial r})dr={2\pi (r\frac{\partial C}{\partial r})|}_{r=0}^{r=R}=2\pi R{(\frac{\partial C}{\partial r})}_{r=R}.$$

Consequently, the integration of the oxygen transport equation, which does not involve the relationship between hemoglobin concentration and oxygen transport, over the cross-section of the blood vessel is given as follows:

5 $$\rho \frac{\partial M}{\partial t}+\rho {v}_{z}\frac{\partial M}{\partial z}=2\pi R{({\rm{\Gamma }}\frac{\partial C}{\partial r})}_{r=R}+{\rm{\Gamma }}\frac{{\partial }^{2}M}{\partial {z}^{2}},$$

where the *z* direction is the direction of blood flow. Although the effect of hemoglobin is not considered in the oxygen transport equation, the oxygen content numerically predicted by considering the oxyhemoglobin transport can be calculated according to the numerical simulation of oxygen transport without consideration of hemoglobin. It was found that the oxygen content resulted from oxygen transport with hemoglobin had an approximately linear relationship with the numerical results of the oxygen transport equation without considering hemoglobin^4^.

The convective flux is expressed as *J*_*con*_ = *ρv*_*z*_*M*, and the diffusive flux is expressed as ${J}_{diff}={\rm{\Gamma }}\frac{\partial M}{\partial z}$. Moreover, *x* generally represents the direction of the fluid flow in the fluid transmission line equations. In the following equation, *z* is replaced by *x* according to the fluid transmission line equations. Therefore, equation (5) should be transformed into:

6 $$\frac{\partial }{\partial t}(\frac{{J}_{con}}{{v}_{x}})+\frac{\partial }{\partial x}({J}_{con})=\frac{\partial }{\partial x}({J}_{diff})+2\pi R{({\rm{\Gamma }}\frac{\partial C}{\partial r})}_{r=R}.$$

The three following assumptions are applied in the derivation of the above equation: the vessel is treated as a uniform tube; the velocity distribution is uniform over the entire cross-section of the vessel; and the value of the vascular radial deformation is much smaller than the radius. These assumptions were also employed in the derivation of the fluid transmission line equations^24–26^.

### Transmission line equations of oxygen transport

In mass transport, the solute concentration distribution is mainly influenced by the convective flux and the diffusive flux. Moreover, a correlation exists between the convective flux and the diffusive flux^27^. The convective flux could increase the oxygen concentration gradient, which then enhances the diffusive flux^28,29^. In contrast, diffusion is supposed to affect the convective flow, which dominates the convective flux^30^. A similar relationship also exists between the convective flux and the diffusive flux in oxygen transport^12^. Moreover, the heat generated by convection is proportional to the gradient of the thermal diffusion in the heat transfer of a plane coquette fluid flow^31^. In this study, the fluid transmission line equations $$(\{\begin{array}{c}-\frac{\partial P}{\partial x}={Z}_{x}Q\\ -\frac{\partial Q}{\partial x}=\frac{1}{{Z}_{r}}P\end{array})$$ are regarded as the theoretical reference for developing the transmission line equations of oxygen transport, where *P* is the pressure; *Q* is the volumetric flow rate; *Z*_*x*_ is the transverse impedance; and *Z*_*r*_ is the longitudinal impedance. The parameters in the fluid transmission line equations are derived from the analogy between the governing equations of fluid flow and the electrical transmission line equations. And these parameters could be adjusted on the basis of different simplifications and mathematical treatments of the continuity equation and the Navier-Stokes equations. In terms of blood flow in an artery, physiological and physical properties are involved in the development of the transmission line equations of blood flow. The lumped parameter model of blood flow is derived from the integration of the transmission line equations along the vessel in each uniform vascular segment, and partial differential equations of the transmission line model are converted into ordinary differential equations of the lumped parameter model after this integration.

The following transmission line equations of oxygen transport are developed as follows:

7 $$-\frac{\partial {J}_{diff}}{\partial x}/{J}_{con}={Z}_{con}$$

8 $$-\frac{\partial {J}_{con}}{\partial x}/{J}_{diff}=Z{}_{diff},$$

where the ratio of the gradient of the diffusive flux to the convective flux is defined as the convective impedance and denoted as *Z*_*con*_, while the ratio of the gradient of the convective flux to the diffusive flux is defined as the diffusive impedance and denoted as *Z*_*diff*_.

In oxygen transport, diffusion is mainly influenced by convection^32^, while convection is dominated by blood flow^27^. Thus, diffusion rarely has an effect on convection^30,31,33,34^. The following relationships were determined based on the mass balance of oxygen transport in a vessel: convective transport = diffusive flux out of the blood = oxygen consumption^2^. Consequently, equation (8) could be rewritten as follows:

9 $$-\frac{\partial {J}_{con}}{\partial x}={-{J}_{diff}|}_{{\rm{From}}{\rm{blood}}{\rm{to}}{\rm{wall}}}=-2\pi R{({\rm{\Gamma }}\frac{\partial C}{\partial r})}_{r=R},$$

where $-2\pi R{({\rm{\Gamma }}\frac{\partial C}{\partial r})}_{r=R}$ represents the diffusive flux of oxygen from the blood to the vascular wall which is the major source for supplying oxygen to the avascular zone of the arterial wall^35^.

A phase difference exists between the pressure gradient and the volumetric flow rate. Hence, one of the fluid transmission line equations could be written as:

10 $$-\frac{\partial P}{\partial x}={Z}_{X}Q=R^{\prime} Q+L^{\prime} \frac{\partial Q}{\partial t},$$

where *R*′ and *L*′ represent resistivity and inertance, respectively. A phase difference also exists between the gradient of the diffusive flux and the convective flux. Thus, equation (7) can be rewritten as:

11 $$-\frac{\partial {J}_{diff}}{\partial x}={R}_{0}\,{J}_{con}+{L}_{0}\frac{\partial {J}_{con}}{\partial t}.$$

In summary, the transmission line equations of oxygen transport in a vessel are developed as follows:

12 $$\{\begin{array}{c}-\frac{\partial {J}_{diff}}{\partial x}={R}_{0}\,{J}_{con}+{L}_{0}\frac{\partial {J}_{con}}{\partial t}\\ -\frac{\partial {J}_{con}}{\partial x}={-{J}_{diff}|}_{{\rm{Frombloodtowall}}}=-2\pi R{({\rm{\Gamma }}\frac{\partial C}{\partial r})}_{r=R}\end{array},$$

where *R*_0_ and *L*_0_ represent the properties of oxygen convection, which are elucidated in the section that follows.

### Derivation of the expressions for *R*_0_ and *L*_0_

The oxygen transport equation (equation (6)) can be rewritten as:

13 $$\frac{1}{{v}_{x}}\frac{\partial {J}_{con}}{\partial t}-\frac{1}{{{v}_{x}}^{2}}\frac{\partial {v}_{x}}{\partial t}\cdot {J}_{con}+\frac{\partial {J}_{con}}{\partial x}=\frac{\partial {J}_{diff}}{\partial x}+{{J}_{diff}|}_{{\rm{From}}{\rm{blood}}{\rm{to}}{\rm{wall}}}.$$

Subsequently, subtracting equation (9) from equation (11) yields:

14 $$-\frac{\partial {J}_{diff}}{\partial x}+\frac{\partial {J}_{con}}{\partial x}={R}_{0}\,{J}_{con}+{L}_{0}\frac{\partial {J}_{con}}{\partial t}+{{J}_{diff}|}_{{\rm{From}}{\rm{blood}}{\rm{to}}{\rm{wall}}}.$$

Comparing equation (13) and equation (14), *R*_0_ and *L*_0_ can be provided by the following expressions:

15 $$\{\begin{array}{c}{R}_{0}=\frac{1}{{{v}_{x}}^{2}}\frac{\partial {v}_{x}}{\partial t}=\frac{\partial }{\partial t}(-\frac{1}{{v}_{x}})\\ {L}_{0}=-\frac{1}{{v}_{x}}\end{array}.$$

The interval transit time of mass transport is defined as the reciprocal of the velocity^36–38^. Hence, *L*_0_ represents the transit time of oxygen per unit distance. Moreover, the first derivative of transit time versus time is equal to 1 − *H*(*t*), where *H*(*t*) is the cumulative frequency function of transit time and represents the fraction of oxygen transmission^36,39^. *R*_0_ represents the fraction of the remaining oxygen convection per unit distance. Both *L*_0_ and *R*_0_ belong to the characteristics of oxygen convection in oxygen transport of a vessel. Specifically, *L*_0_ and *R*_0_ are supposed to represent the inertance and resistance of the convective flow to the oxygen transport in each vascular segment, respectively. *L*_0_ and *R*_0_ are the components of the convective impedance which determines the relationship between the gradient of the diffusive flux and the convective flux.

Finally, the transmission line equations of oxygen transport are provided by the following expressions:

16 $$\{\begin{array}{c}-\frac{\partial {J}_{diff}}{\partial x}={R}_{0}\,{J}_{con}+{L}_{0}\frac{\partial {J}_{con}}{\partial t}\\ -\frac{\partial {J}_{con}}{\partial x}={-{J}_{diff}|}_{{\rm{From}}{\rm{blood}}{\rm{to}}{\rm{wall}}}=-2\pi R{({\rm{\Gamma }}\frac{\partial C}{\partial r})}_{r=R}\end{array},$$

where,

17 $$\{\begin{array}{c}{R}_{0}=\frac{1}{{{v}_{x}}^{2}}\frac{\partial {v}_{x}}{\partial t}=\frac{\partial }{\partial t}(-\frac{1}{{v}_{x}})\\ {L}_{0}=-\frac{1}{{v}_{x}}\end{array}$$

where *L*_0_ and *R*_0_ are the transit time of oxygen per unit distance and the fraction of remaining oxygen convection per unit distance, respectively.

## Verification

Three 3D fluid–structure interaction (FSI) models coupled with the oxygen transport equation and the corresponding lumped parameter models of oxygen transport were employed to numerically verify the developed transmission line equations of oxygen transport.

**Figure 1:**
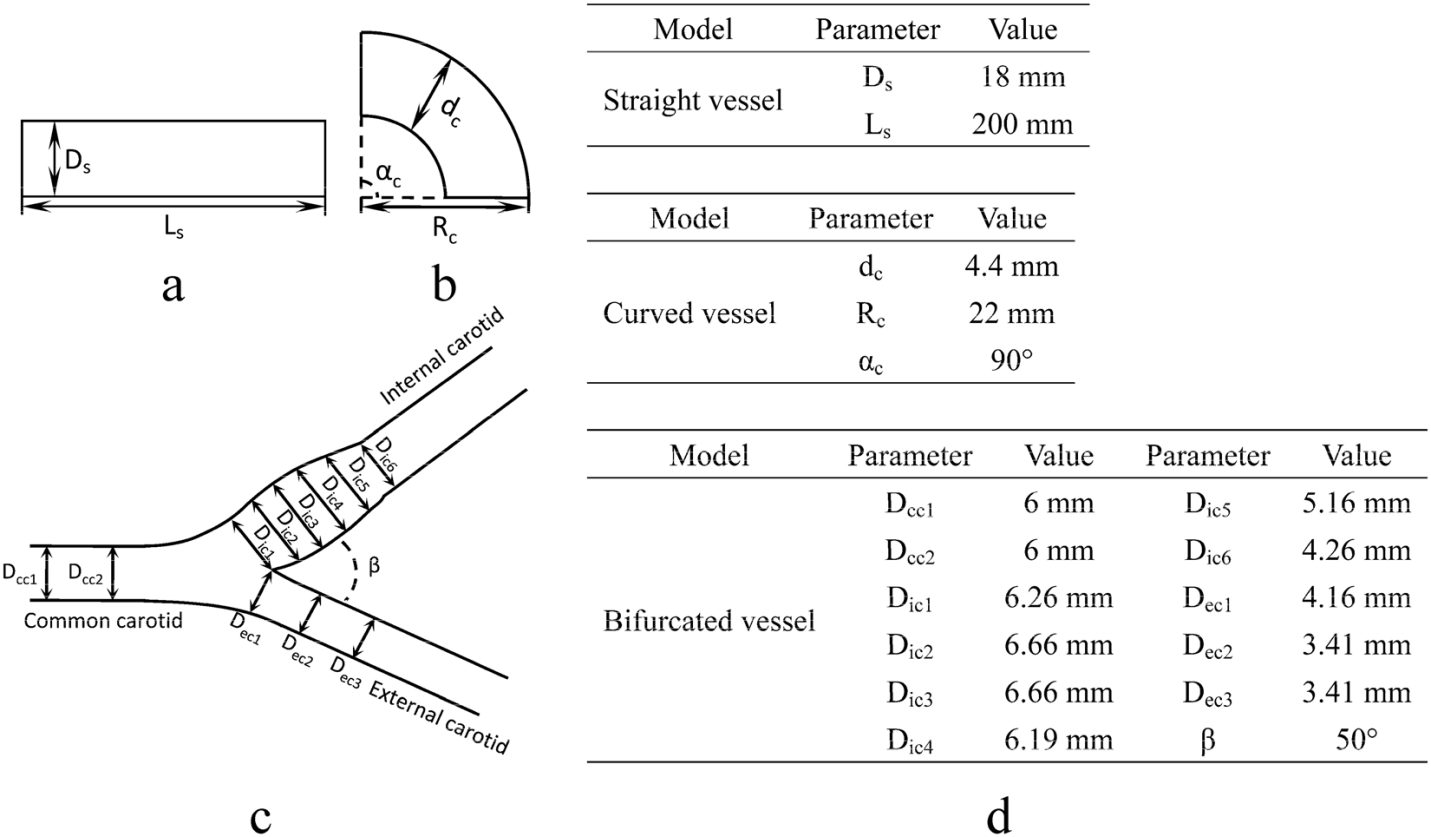
Schematic diagrams of the geometrical models of (**a**) straight vessel, (**b**) curved vessel and (**c**) bifurcated vessel. (**d**) Geometrical parameters of the vessels. D_s_ and L_s_ represent the diameter and the length of the straight vessel; d_c_, R_c_ and α_c_ represent the diameter, the radius of curvature and the angle of curvature of the curved vessel, respectively; D_ccl_ (l = 1, 2), D_icm_ (m = 1, 2, 3, 4, 5, 6), D_ecn_ (n = 1, 2, 3) and β represent the diameters of the common carotid artery, the diameters of the internal carotid artery, the diameters of the external carotid artery and the bifurcation angle of the bifurcated vessel, respectively.

**Figure 2:**
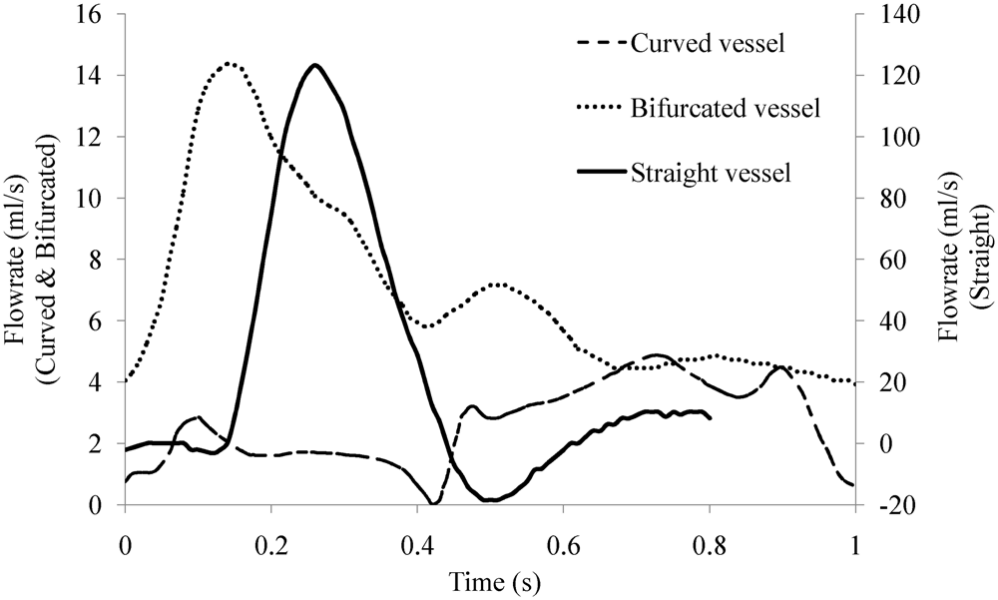
Flowrates at the inlets of three tubes.

In the FSI analyses, the geometrical models and parameters of three vessels (straight vessel, curved vessel and bifurcated vessel) were shown in Fig. 1. The geometrical models of these three vessels are the most common and fundamental in the circulatory system. And the parameters used in the straight vessel, the curved vessel and the bifurcated vessel were referred to the physical properties of the abdominal artery, the coronary artery and the carotid artery, respectively. Since understanding the oxygen concentration distribution played a significant role in the assessment and treatment of the diseases in these arteries, most of studies about oxygen transport in vessels have focused on the prediction of the oxygen concentration distribution in the abdominal artery, the coronary artery and the carotid artery^14,40–42^. The governing equations of blood flow were the 3D Navier-Stokes equation and the continuity equation. In terms of the transient structural simulation, the momentum balance equations were solved with a fluid–solid interface boundary and constraint conditions. The mass transport equation was used to describe oxygen transport in blood. The density and the dynamic viscosity of blood were set as 1050 kg/m^3^ and 3.5 × 10^−3^ kg/(m·s)^43^, respectively. The values of the tube wall thicknesses were 2 mm, 0.18 mm and 0.7 mm^40,44,45^, respectively. And the elastic moduli of the walls were set as 5.11 MPa, 0.7 MPa and 0.5 MPa^40,46^, respectively. The density and Poisson’s ratio of the wall were 1060 kg/m^3^ and 0.49. The diffusion coefficient of oxygen in blood was 1.2 × 10^−9^ m^2^/s^47^. In terms of blood flow, the pulsatile profiles of the flowrates were applied at the inlets of three vascular models according to *in-vivo* measurements (Fig. 2)^14,40,48^. Three pressure boundary conditions coupled with the constant resistance models were applied at the outlets, and the values of the resistances were 0.0865 mmHg·s/cm^3^, 55.6 mmHg·s/cm^3^, 35.3 mmHg·s/cm^3^ (External carotid, EC) and 21.7 mmHg·s/cm^3^ (Internal carotid, IC)^49,50^, respectively. In terms of oxygen transport, the values of oxygen tensions at the inlets were 85 mmHg, 85 mmHg and 85.3 mmHg, whereas those at the inner surfaces of the vascular walls were specified as 60 mmHg, 48 mmHg and 55.5 mmHg^4,12,14,51,52^. The commercial finite-element package ANSYS (version 14.0, ANSYS, Inc., USA) was used to simulate blood flow and oxygen transport. Three user-defined C-like functions (UDFs) were also compiled in setting the inlet and outlet boundary conditions.

**Figure 3:**
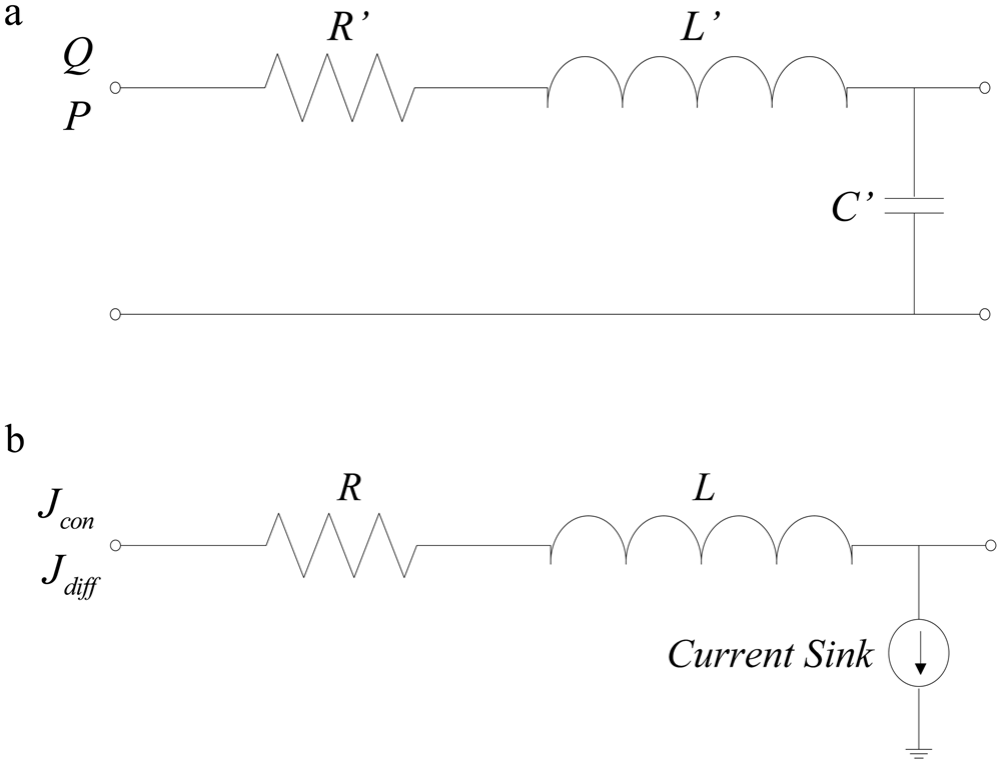
Lumped parameter representations of a basic segment. (**a**) Electrical analogue of blood flow with resistance (*R’*), inductance (*L’*), and capacitance (*C’*) (*R’*, *L’*, and *C’* represent resistivity, inertance, and compliance, respectively). Current and voltage are related to the flow rate and pressure, respectively. (**b**) Electrical analogue of oxygen transport with resistance (*R*), inductance (*L*), and current sink (*R* and *L* represent the properties of oxygen convection). The current sink is related to the diffusive flux of oxygen from the blood to the vascular wall. Current and voltage represent the convective flux and diffusive flux of oxygen, respectively.

Meanwhile, the corresponding lumped parameter models of blood flow and oxygen transport in these tubes were developed to numerically predict the oxygen concentration distribution. First, a numerical analysis of the lumped parameter model of blood flow was conducted to provide the blood velocity for calculating the parameters (*L*_0_ and *R*_0_) in the lumped parameter model of oxygen transport. In these computational models, the straight tube and the curved tube were divided into ten segments and nine segments, moreover, the main vessel and the branches in the bifurcated tube were respectively divided into ten segments. Each segment in different tubes had a length of 20 mm (Straight)/3.84 mm (Curved)/6.45 mm (Common carotid, CC)/6.23 mm (IC)/6.2 mm (EC), which was short enough to precisely calculate the blood velocity in the lumped parameter model of blood flow^16,53^. The electric circuits related to the transmission line equations of blood flow and oxygen transport were used to represent the lumped parameter models of blood flow and oxygen transport in each segment (Fig. 3). The material properties and the boundary conditions in the lumped parameter models were the same as those of the 3D FSI models. The fourth-order Runge–Kutta method was employed in the numerical investigations of these lumped parameter models.

**Figure 4:**
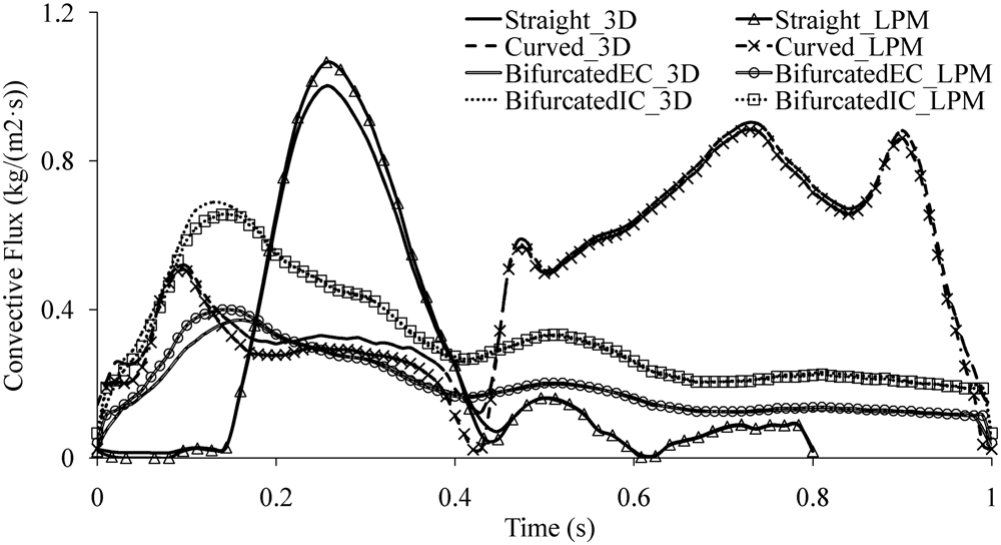
Convective flux of oxygen at the end of each tube.

In summary, the oxygen concentration distributions in the tubes were acquired through the numerical analyses of the 3D FSI models coupled with oxygen transport and the lumped parameter models of oxygen transport. Figure 4 shows that the difference of the oxygen convective flux between the 3D model and the lumped parameter model at the end of each tube was quite modest. Consequently, the lumped parameter model of oxygen transport based on the transmission line equations of oxygen transport could numerically predict the oxygen concentration distribution, which was in accordance with the 3D computational simulation results. According to the comparison between the lumped parameter model and the 3D computational model, the transmission line equations of oxygen transport are reliable.

The oxygen concentration distribution in the vessel mainly depends on hemodynamic responses. The lumped parameter model of blood flow could provide reliable hemodynamic data for complex vascular geometries. Thus, the lumped parameter model of oxygen transport based on the transmission line equations of oxygen transport and the lumped parameter model of blood flow should provide a reliable prediction of the oxygen distribution in the vessels with branching and complex structure. In terms of branching and complex vascular structures, the parameters in the transmission line equations of oxygen transport would be adjusted according to the lumped parameter model of blood flow. A sensitivity analysis of the parameters will be performed in the next stage of our study. The straight vessel, the curved vessel and the bifurcated vessel have been selected in the 3D computational simulations herein to verify the transmission line equations of oxygen transport.

## Discussion

This study introduces a process for deducing the transmission line equations of oxygen transport in a vessel. More specifically, the fluid transmission line equations are regarded as the theoretical reference for establishing the oxygen transmission line equations. The theory of oxygen transport in a vessel, which indicates that the transmitted flux of oxygen convection is equal to the diffusive flux out of the blood, is the theoretical basis for proposing the transmission line equations of oxygen transport. The mathematical expressions of the parameters (*L*_0_ and *R*_0_) in the oxygen transmission line equations are derived based on the mass transport equation. These parameters represent the properties of oxygen convection in oxygen transport. The lumped parameter model of oxygen transport based on the oxygen transmission line equations can be used to provide numerical predictions for the systemic distribution of the oxygen concentration in the arterial tree. A clear understanding of the systemic distribution of the oxygen concentration should be helpful for assessing the status of the human body and for offering valuable guidance for the prevention and intervention of diseases.

The oxygen concentration in blood was estimated in the previous studies based on an empirical formula, which was primarily related to the hemoglobin in terms of the whole circulatory system^21,22^. Moreover, the value of the hemoglobin concentration included in the empirical formula was set as a constant in each part of the circulatory system. The effects of hemodynamics on the oxygen concentration were not considered in the establishment of the previous empirical formulas. However, the hemoglobin and oxygen concentrations are mainly affected by the convective flow in terms of the hemodynamic effects. Thus, the oxygen convection, which is determined by the convective flow, in this study is involved in the development of the transmission line equations of oxygen transport. The parameters in these transmission line equations are provided by the expressions composed of blood velocity. Although the effect of hemoglobin is not directly considered in the development of the oxygen transmission line equations, the oxygen concentration numerically predicted by considering the oxyhemoglobin transport could be derived from the results of the oxygen lumped parameter model developed from these equations. In fact, the oxygen content calculated with oxyhemoglobin transport was approximately linearly related to the numerical results in the CFD simulations without considering hemoglobin^4^.

The systemic distribution of the oxygen concentration could also be acquired from the 3D CFD model of the whole arterial system in addition to the aforementioned numerical methods and our method. However, conducting a 3D simulation comprising the CFD analysis and the mass transport computation for the entire arterial system requires a large number of computational resources and consumes much time^54,55^. Moreover, the advantages of the proposed lumped parameter model are presented in two aspects: in mathematics, partial differential equations are converted into ordinary differential equations, which simplifies the numerical analysis; in physics, the complex vascular network is divided into a few lumped parameter elements, while it requires a large number of grid elements to acquire accurate results in the 3D simulation. Thus, the numerical analysis of the oxygen lumped parameter model should require fewer computational resources and much less time in comparison with the 3D simulation, which further demonstrates the significance and value of these transmission line equations of oxygen transport.

In addition to oxygen, Acquiring the concentration distribution of other substances in the entire vascular system could provide guidance for the assessment and intervention of vascular disease (e.g., contrast agents for vascular imaging, low-density lipoprotein (LDL), and targeted drugs). More specifically, determining the systemic distribution of contrast agent concentration is helpful for optimizing vascular imaging quality^56–58^. LDL accumulation is a risk factor for the atherosclerosis development in an artery^42,59^. Thus, determining the systemic distribution of the LDL concentration in the arterial system is beneficial in assessing the atherosclerotic development. Numerous targeted drugs also have a detrimental effect on normal tissues because of their toxicities. Therefore, understanding drug delivery in blood vessels is beneficial in improving targeted drug therapies^60–62^. The systemic distribution of the substance concentration can be obtained from numerical investigations of these lumped parameter models with reduced computational costs and time. The transmission line equations of oxygen transport established in this manuscript could be used as a theoretical reference for developing lumped parameter models of the delivery of these substances in blood. These transmission line equations should be modified according to the critical characteristics of the corresponding substance delivery. First, the source terms in the mass transport equations should be adjusted based on their biological and chemical properties in blood to consider the biological and chemical interactions between these substances and the blood. Subsequently, the mass transport equation with the adjusted source term would be integrated over the cross-section of the vessel under some assumptions. The integrated form of the mass transport equation is transformed into the equation including the diffusive flux and the convective flux. The diffusive flux of each substance is adjusted by substituting the appropriate diffusion coefficient because the diffusion coefficients differ for different substances in blood. Second, the basic transmission line equations of the corresponding substance are proposed according to the physiological and physical properties of the substance delivery. The expressions of the parameters in the proposed transmission line equations of other substances are acquired through the simplifications and the mathematic treatments of the basic transmission line equations and the integrated forms of the mass transport equations. More specifically, the values of the parameters in the transmission line equations are supposed to depend on blood flow.

Although these novel transmission line equations of oxygen transport are important, a few limitations exist. First, more physiological properties of oxygen in blood should be involved in the continued development of these equations to obtain a more precise estimation of the oxygen concentration distribution in the arterial system. Second, the diffusive flux of oxygen from the blood to the arterial wall dominates the distribution of the convective flux in blood, and obtaining the value of the diffusive flux into the arterial wall is difficult. The oxygen consumption in the arterial wall could generally be regarded as an estimation of the diffusive flux into the wall, which could affect the numerical prediction of the oxygen concentration in the lumped parameter model of oxygen transport. A noninvasive method for measuring the gradient of the oxygen concentration near the arterial wall is beneficial for acquiring an accurate value of the oxygen flux into the wall and can enhance the accuracy of the oxygen concentration prediction. In terms of the whole circulatory system, the number of the lumped parameter elements and oxygen transport in the terminal microvasculature should be emphasized in the establishment of the lumped parameter model of oxygen transport. The number of the lumped parameter elements significantly affects the numerical results of the lumped parameter models of blood flow and oxygen transport, and acquiring the precise numerical results of the lumped parameter model requires sufficient lumped parameter elements. Moreover, the distal boundary conditions at the ends of the lumped parameter model of the whole circulatory system mainly depend on the oxygen consumption and blood velocity in the microcirculation.

### Data availability statement

All data generated or analysed during this study are included in this published article.
